# Illicit COVID-19 products online: A mixed-method approach for identifying and preventing online health risks

**DOI:** 10.1371/journal.pone.0287231

**Published:** 2023-06-16

**Authors:** Valeria Catalani, Honor D. Townshend, Mariya Prilutskaya, Robert P. Chilcott, Antonio Metastasio, Hani Banayoti, Tim McSweeney, Ornella Corazza

**Affiliations:** 1 School of Life and Medical Sciences, University of Hertfordshire, Hatfield, United Kingdom; 2 School of Law, University of Hertfordshire, Hatfield, United Kingdom; 3 Department of Personalised Medicine, Pavlodar Branch of Semey Medical University, Pavlodar, Kazakhstan; 4 Cybersolace Limited, London, United Kingdom; 5 Department of Psychology and Cognitive Science, University of Trento, Rovereto, Italy; Non-Communicable Diseases Research Center, Endocrinology and Metabolism Research Institute, Tehran University of Medical Sciences, ISLAMIC REPUBLIC OF IRAN

## Abstract

**Aims:**

The COVID-19 pandemic triggered a demand for vaccines, cures, and the need of related documentation for travel, work and other purposes. Our project aimed to identify the illicit availability of such products across the Dark Web Markets (DWMs).

**Methods:**

A retrospective search for COVID-19 related products was carried out across 118 DWMs since the start of the pandemic (March 2020-October 2021). Data on vendors as well as advertised goods such as asking price, marketplace, listed date were collected and further validated through additional searches on the open web to verify the information relating to specific marketplaces. Both quantitative and qualitative methods were used for data analysis.

**Results:**

Forty-two listings of unlicenced COVID-19 cures and vaccination certificates were identified across 8 marketplaces sold by 25 vendors with significant variation in prices. The listings were found to be geographically specific and followed the progression of the pandemic in terms of availability. Correlations between vendor portfolios of COVID-19 products and variety of goods of other illicit nature such as illegal weaponry, medication/drugs of abuse also emerged from our analysis.

**Conclusion:**

This study is one of the first attempts to identify the availability of unlicenced COVID-19 products on DWMs. The easy accessibility to vaccines, fake test certificates and hypothetical/illegal cures poses serious health risks to (potential) buyers due to the uncontrolled nature of such products. It also exposes buyers to an unwanted contact with vendors selling a variety of other dangerous illicit goods. Further monitoring and regulatory responses should be implemented to protect the health and safety of citizens especially at times of global crisis.

## Introduction

The dark web, sometimes referred to as the ‘onion web’, is an internet-accessible space offering prohibited items for sale on marketplaces [1]. Though most associated with the illicit drug market [2], dark web marketplaces (DWMs) provide an unregulated platform for the sale and purchase of any item (listings). Recent data report how two thirds of the available dark web listings, are represented by drug related items, including newly developed or previously unknown chemical and pharmaceutical, such as novel psychoactive substances [3, 4]. Next to these, listings as falsified documents, malware, fraud products, weapons, protected wildlife and human organs can be found, often from the same vendor portfolio [5]. DWMs are highly volatile and dynamic in nature: new products are added or removed to crime portfolio according to market demand, with criminal businesses becoming active in pursuit of newly emerging profit opportunities [5]. To ensure anonymity of both sellers and vendors and non-traceability of payments, transactions are made with cryptocurrencies, such as Bitcoins or Monero, facilitated by automatic encryption via the usage of Pretty Good Privacy cryptography [6]. This very volatile nature was confirmed during the COVID-19 pandemic, when the disruption, spread of misinformation and confusion caused, were quickly capitalised upon by criminal groups looking to expand their business portfolio with the sale of counterfeit antiviral medications, COVID-19 vaccines, and other medical products [7–10]. The surge in demand for vaccines and certifications as part of various government measures to facilitate freedom of movement and access to services for individuals [11–14] in combination with the shortages of supply observed for these products in various geographical areas [15], made their e-commerce even more appealing [16]. In addition, the demand for cures saw an increase due to the infodemic and misinformation about the risks and the number of COVID-19 cases happening during the pandemic [17]. The purpose of this study was to assess availability of illicit COVID-19 vaccines and related goods on DWMs at the start of the pandemic.

## Methods

### Study design and data sources

Digital trace data was collected from prominent darknet marketplaces, using a secured platform provided by the cybersecurity company CyberSolace, a private sector company specialising in information security advisory services. The platform is primarily used for threat intelligence and provides monitoring of the DWMs by performing rapid and reproducible searches for different marketplaces, vendors, and items. The ‘search spiders’ developed using Python scripts allow a rapid scanning of the darknet as well as a retrospective analysis of the marketplaces which no longer exist due to law enforcement operation or exit scams. This is an advantage compared to Tor or other traditional browsers which are unable to detect activities of markets that are no longer available. Searches were conducted in October 2021 and covered the period from March 2020 to October 2021. This timeframe was chosen as inclusive of the start of COVID-19 pandemic and the introduction of restrictions worldwide. The keywords used were identified in relation to a) COVID-19 pandemic (e.g., Coronavirus; COVID; COVID19; COVID-19; Corona; Rona); b) product types (Pass, Vaccine, Vaccination, Card, Cure, Certificate, Medicine); specific vaccine brand names (e.g., Moderna); and other related keywords (e.g., ventilator) and colloquialisms (e.g., covaxin). Duplicates, either on the same thread or from previously observed threads, were removed from the final hits. The data collected for each related search term were classified according to a) listing title b) product type c) description of items sold d) asking price e) marketplace f) vendor g) Bitcoin wallet details h) vendor name and contact methods i) listed date and j) any additional description about the vendor available. For the scope of the paper private details on the vendors have been removed. Results were manually filtered and then validated through searches on darknet dedicated online sources to verify the information relating to specific marketplaces. Further information on classification methods is available on Table 1. No participant’s consent was needed for this study, because information was freely available on the darkweb.

**Table 1:** List of product types adopted for hits categorisation. QR stands for Quick Results.

COVID-19 related product types	
**Vaccines**	Moderna, unspecified vaccines
**Cures**	Illicit and legitimate medications/substances
**Certificates**	Travel passes, vaccination certifications blank vaccine cards, QR code vaccination passes
**Tests**	Rapid tests, antibody tests, self-tests
**Medical supplies**	Masks, respirators

### Data analysis

Results were analysed using both qualitative (e.g., posts time frame, vendors reported geographical area, price, and vendor’s profile) and quantitative methodologies. The possible correlates between the sale of COVID-19 and non-COVID-19 related items were also considered using a descriptive-correlational design. The structure of the products was first described in frequencies (%) according to the product types, with Spearman correlation coefficients (r) subsequently calculated.

### Ethics

This study was approved by the Human Sciences Ethics Committee at the University of Hertfordshire, protocol number: aLMS/SF/UH/02951(2) as well as by the funding partner’s Ethics committee.

## Results

### Qualitative analysis

A total of 42 listings for COVID-19 related products were identified across the 118 DWMs. These were sold across eight marketplaces by 25 vendors as presented in Table 2.

**Table 2:** Qualitative analysis dataset (the products are listed in orded of search term).

Search Term	Listing Title	Product type	Vendor	Location	Marketplace	Asking price	Posting Date
COVID19	COVID-19 vaccine registration (nl residents only)	Certificate	1	Netherlands	Dark0de	1,800 €	05/10/2021
COVID19	CDC vaccination certificate card	Certificate	2	United States	Dark0de	55 €	05/10/2021
COVID19	[DD] vaccination certificate for sale with QR code	Certificate	3	Worldwide	Dark0de	300 $	14/10/2021
COVID19	COVID-19 registration (NL Residents only, working QR app)	Certificate	1	Netherlands	Versus	1,500 €	23/10/2021
COVID19	Health Pass / Covid Pass / Green Pass / Pass Sanitaire	Certificate	4	Europe	Cartel	405 $	11/10/2021
COVID19	COVID vaccine digital certificate	Certificate	5	USA	Dark0de	100 $	29/10/2021
COVID19	Buy High Quality Real Documents (Passport, Id, Dl, Visas, Birth Certificates, Residence Document,….., COVID Cards)	Certificate	6	Worldwide	Cartel	1200 $	20/09/2021
COVID19+certificate	buy your authentic and registered Passports, Drivers Licenses, ID Cards, COVID19 Vaccine Cards, Birth Certificate, Fake Bills And Related Documents Online	Certificate	7	Europe	Cartel	3029.25 $	09/06/2021
Covid+vaccine	COVID19 Impfbuch Impfpass Certificate Vaccination Corona	Certificate	8	Europe	WhiteHouseMarket	1 €	20/09/2021
COVID19 +vaccine	COVID-19Vaccination Record Card USA CDC authentic	Certificate	9	USA	Dark0de	100 €	01/09/2021
COVID19 +vaccine	CDC Vaccination Certificate For Sale	Certificate	3	Worldwide	Dark0de	300 €	09/09/2021
COVID19 +vaccine	COVID-19Vaccine Passes | Fake Negative Papers | COVID-19Fit To Travel Certificates	Certificate	10	France and Europe	Cartel	470 €	28/09/2021
COVID19 +vaccine	Vaccination Card Certificate For Sale	Certificate	3	Europe	Dark0de	300 €	01/09/2021
COVID+test	EU Digital Covid Certificate	Certificate	11	Worldwide	Dark0de	1,950 €	24/09/2021
COVID19	Steroid for COVID-19	Cure	12	USA	Cartel	1.50 $	05/07/2021
COVID19 +vaccine	Protect yourself from the Corona Virus Caps (20 capsules)	Cure	13	Europe	Europa	40 €	21/04/2020
COVID+treatment	Iverheal 6mg (Ivermectin) COVID-19treatment—100 tabs	Cure	14	India	WhiteHouseMarket	43 $	09/09/2021
COVID+treatment	Amantadine Amantrel 100 mg (potential Covid treatment) 60/120/450 tabs	Cure	15	India and Singapore	WhiteHouseMarket	80/130/199 $	09/09/2021
COVID+treatment	Ivermectin 6 mg Stromectol (COVID-19treatment) 50/100/200 tabs	Cure	16	India	WhiteHouseMarket	24/44/77 $	16/08/2021
COVID+treatment	Iverheal 6 mg (Ivermectin—COVID-19treatment) 100 pills	Cure	16	India	WhiteHouseMarket	38 $	16/08/2021
COVID+treatment	Iverheal 12mg (Ivermectin—COVID-19treatment) 100 pills	Cure	16	India	WhiteHouseMarket	50 $	16/08/2021
COVID+treatment	Iverheal 12mg (COVID-19treatment) 100-200-500 pills	Cure	15	India and Singapore	Dark0de	199 $	09/08/2021
COVID+treatment	Iverheal 12mg (COVID-19treatment) 100-200-500 pills	Cure	15	India and Singapore	WhiteHouseMarket	199 $	02/08/2021
COVID+treatment	Ivermectin 6mg Stromectol (COVID-19treatment) 50/100/200/500 tabs	Cure	17	India	WhiteHouseMarket	25/45/79/169 $	02/08/2021
COVID+treatment	5gr CORONA PACK—5 gr Calm Weed for 30$ (no track)	Cure	18	India and Singapore	Cannazon	30.61 €	06/04/2020
COVID+treatment	HCQ hydroxychloroquine 200mg 10 tablet pack	Cure	19	USA	Dark0de	40 $	04/05/2021
COVID+treatment	HCQ Hydroxychloroquine 200mg 10 tablet pack	Cure	19	USA	DarkMarket	50 $	21/10/2020
Corona	Corona Anti-Virus Face Mask Ready and, Gowns	Medical Supplies	20	USA	Europa	111 €	27/03/2020
COVID+vaccine	Get solution the COVID-19 vaccine full dose	N.A.	N.A.	N.A.	Cartel	£300	25/01/2021
COVID+test	Self-test covid created by La Roche	Test	21	Switzerland	Versus	48 €	05/07/2021
COVID+test	COVID-19 Antibody Test Kit	Test	22	Worldwide	DarkMarket	43.31 $	05/10/2020
COVID+test	25 pcs COVID-19 (coronavirus) quick test	Test	23	Singapore	Versus	366 €	28/07/2020
Corona+vaccine	HELLO buy fast … Corona-Virus Vaccine. Is Out Now	Vaccine	24	NA	Yakuza	200 $	05/07/2020
Moderna+Vaccine	Buy 10 Moderna (mRNA-1273) Covid-19 Vaccine 2000$	Vaccine	25	Worldwide	Cartel	2000 $	06/04/2021

Notes. For formatting puproses a common style (which may differ from the style of the original) has been used for the listing title. CDC stand for Centers for Disease Control and Prevention. Also the name of the vendor was anonymised for privacy purposes.

The same listings (item description, date of posting, prices) were found on multiple DWMs by the same seller, presumably with the aim of reaching different geographic locations. These listings were considered as duplicates and removed. Some of the products identified differed only in quantity and/or price, hence these were grouped together within a single entry. No bitcoin wallet information was retrieved due to the nature of the platform used. In terms of product categories (Table 1), there were 20 “cure”, 13 “certificate”, 3 “test”, 2 “vaccine”, 1 “medical supply” and 1 unassigned. The “cure” entries were represented mostly by IVERMECTIN/IVERHEAL tablets in two different dosages (6 and 12 mg), followed by hydroxychloroquine sulphate tablets (200 mg) and amantadine tablets (Amantrel, 100 mg). A “weed calm package” was also advertised as a cure for COVID-19. The “certificate” category included a variety of products from simple/blank official certificate cards to legitimate vaccination registrations including a functional QR code. These were found to be region restricted (e.g., USA, Netherlands). Only two hits were retrieved for vaccines; Moderna was identified as the manufacturer through the item’s description. Among the hits retrieved for “tests” were COVID-19 tests by La Roche. Where available, the description for each item identified on the dark web is reported in Supplementary Material.

**Fig 1:**
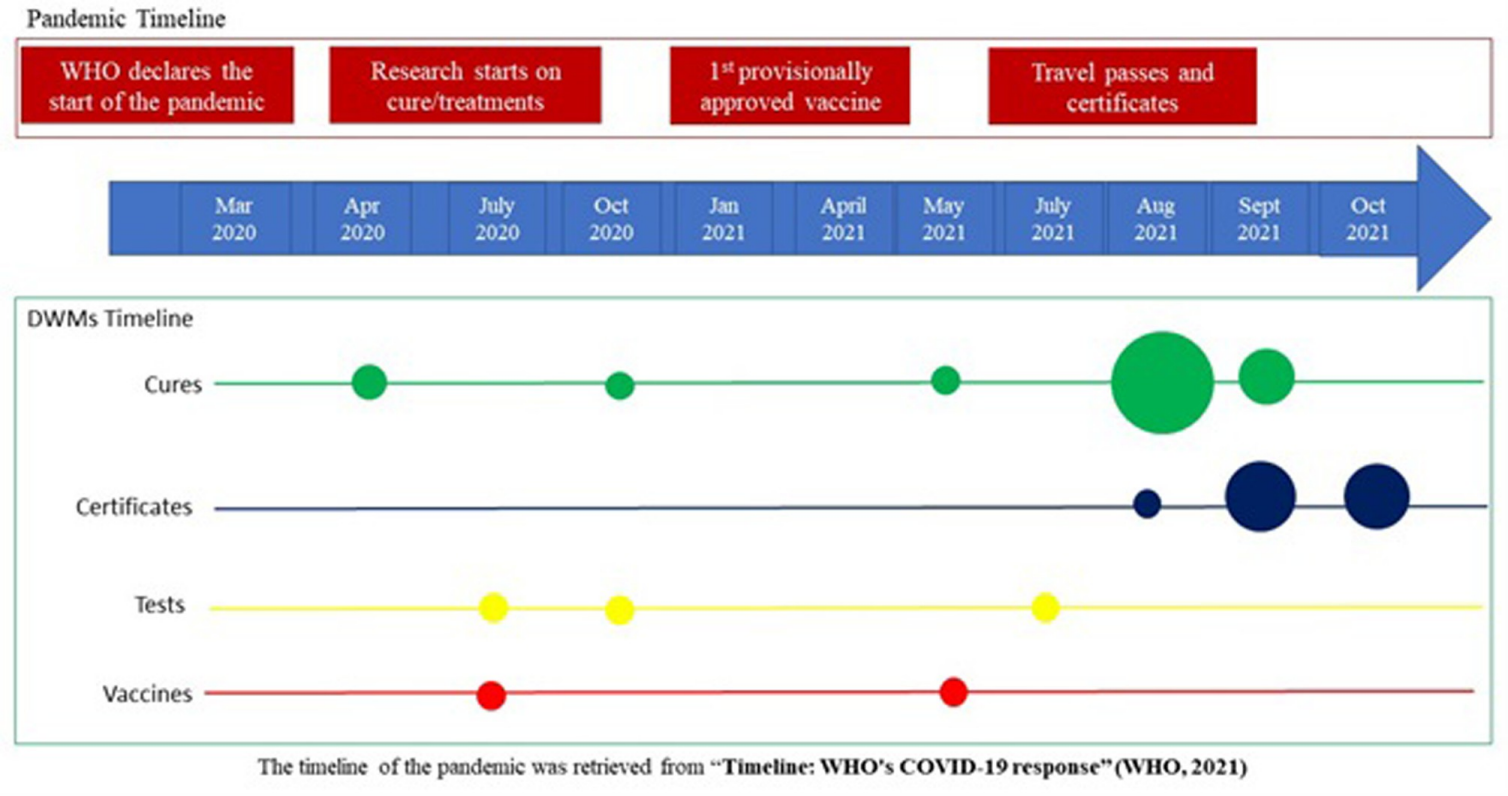


The prices identified across these 40 entries (Table 2) varied from €1 to US$3029. Within the “cure” category, prices were relatively consistent and increased according to dosage (e.g., 6 and 12 mg) and quantity (e.g., 50, 100, 200), with the highest price (US$199) observed for 450 amantadine tablets and 500 IVERHEAL 12 mg pills. Prices associated with the “certificate” product type varied significantly from US$55 for blank vaccine cards to €1500 –US$3200 for official, QR-coded European or USA cards. “Tests” advertised on the dark web had an average price of US$45, which decreased for bulk purchases. The only two hits obtained for vaccines were found to be priced at US$ 200 a shot, independent of the quantity purchased. The COVID-19 related products were advertised in specific time frames that followed the pandemic timeline (Fig 1) The majority of hits retrieved were advertised on the dark web in 2021. All the “certification” hits were advertised starting from June 2021 to October 2021, while dates associated with the “cure” results spanned a broader time space from April 2021 to July 2021. Only one “cure” was advertised earlier (October 2020). Self-tests for COVID-19 were advertised in 2020 only (July and November), while each of the two vaccines were advertised in July 2020 and April 2021.

The COVID-19 related products identified (Table 2) were mostly advertised for sale across specific countries or continents. This was particularly true for “certificate” products, which differed according to geolocation and were country specific. These were mostly for sale in the USA and Europe (Netherlands and France). By contrast, “cure” and “vaccine” products had a wider market presence and were mostly for sale worldwide, originating mainly from India, Singapore, Switzerland and the USA.

The eight DWMs selling COVID-19 related products were Cartel, Cannazon, Dark0de Reborn, Versus, WhiteHouseMarket, Yakuza, Europa and DarkMarket. At the time of the search all were active, except Europa and DarkMarket. The analysis of the content of inactive markets was possible, as mentioned in the method section thanks to the nature of the scraping platform. The number and size of the markets varied from 595 items and 119 vendors (Dark0de Reborn) to ~70,000 items and 2,000 vendors (DarkMarket) (Table 3).

**Table 3:** Marketplaces identified as selling platform for COVID-19 related product.

Marketplace	No. sellers	No. items for sale	COVID-19 Listings
Europa	119	595	1
Dark0de	651	7,055	10
Cartel	439	7,971	8
Cannazon	358	10,312	1
Yakuza	197	10,593	1
Versus	1015	28,510	3
WhiteHouseMarket	1930	49,684	16
Darkmarket	2000	70,000	2

The size of the 25 sellers’ portfolios varied conspicuously as well. The biggest seller (Vendor 22) displayed a portfolio with 872 items on sale, while the smaller (Vendor 4), with only one item on sale. All the vendors were found to be active within the time frame of the search.

### Quantitative analysis

Overall, 3,089 products were retrieved for 25 vendors and classified in 12 different product types (Table 4). Of these, 1.4% (n = 42) were COVID-19 related products (Table 4). Vendor 18 were excluded from the analysis due to absence of any COVID-19-related items: the vendor was identified by the search algorithm due to the disguised sale of an illegal drug as a COVID-19 treatment. Vendors 5 and 13 were excluded due to the lack of further information on their portfolios. The most prevalent listing identified among the product types were medications (including antibiotics, steroids), with 1852 items (60% of all the sold products). Narcotics and unlicensed drugs were offered half as often as medications with 862 items (28%). Money-related products and financial services were identified in 163 cases (5%). Documentation and weapons were found in 2.3% and 1.9% respectively. Table 4 depicts the structure of the product types by vendors.

**Table 4:** Structure of product type by vendors.

*Vendor title*	Medication	Narcotics	Money	Documents	Weapons	Technology	Other	COVID-test	COVID-card	COVID-vaccine	COVID-cure	COVID-mask	Total number	Number of product types
*Vendor 22*	846	0	0	25	0	0	0	1	0	0	0	0	872	3
*Vendor 1*	0	504	0	0	0	0	0	0	2	0	0	0	506	2
*Vendor 15*	105	280	0	0	0	0	0	0	0	0	6	0	391	3
*Vendor 14*	290	10	0	0	0	0	0	0	0	0	1	0	301	3
*Vendor 17*	252	0	0	0	0	0	0	0	0	0	4	0	256	2
*Vendor 24*	44	18	55	1	50	12	0	0	0	1	0	0	181	7
*Vendor 21*	60	13	0	0	0	0	0	1	0	0	0	0	74	3
*Vendor 16*	63	0	0	0	0	0	0	0	0	0	5	0	68	2
*Vendor 19*	65	0	0	0	0	0	0	0	0	0	2	0	67	2
*Vendor 23*	61	0	0	0	0	0	0	1	0	0	0	0	62	2
*Vendor 12*	19	24	12	0	2	0	2	0	0	0	1	1	61	7
*Vendor 25*	0	0	20	12	7	0	13	0	0	3	0	0	55	5
*Vendor 6*	1	12	10	10	0	0	8	0	0	0	0	0	41	5
*Vendor 9*	33	0	0	6	0	1	0	0	1	0	0	0	41	4
*Vendor 11*	0	0	36	2	0	0	1	0	1	0	0	0	40	4
*Vendor 3*	0	0	24	0	0	0	1	0	4	0	0	0	29	3
*Vendor 7*	0	0	0	14	0	0	0	0	3	0	0	0	17	2
*Vendor 2*	11	0	4	0	0	0	0	0	1	0	0	0	16	3
*Vendor 10*	0	1	2	1	0	0	0	0	1	0	0	0	5	4
*Vendor 8*	2	0	0	0	0	0	0	0	1	0	0	0	3	2
*Vendor 4*	0	0	0	0	0	0	1	0	1	0	0	0	2	2
Vendor 20	0	0	0	0	0	0	0	0	0	0	0	1	1	1

In the majority of cases, each vendor dealt with limited product types. Namely, 15 of 22 vendors offered no more than three product types. The highest number of products offered were by Vendor 22 (n = 872) and Vendor 1 (n = 506), which specialized in medications (97%) and illicit drugs (99%). The widest range of products (n = 7) was offered by Vendor 24, which included medications (24%), illicit drugs (10.0%), money (30%), weapons (28%), technologies (67%), documents and COVID-19-vaccines (0.6%); and by Vendor 22 with medications (31%), illicit drugs (39%), money (20%), weapons (3%), COVID-19-masks and COVID-19-cure (1.6%) being listed.

**Fig 2:**
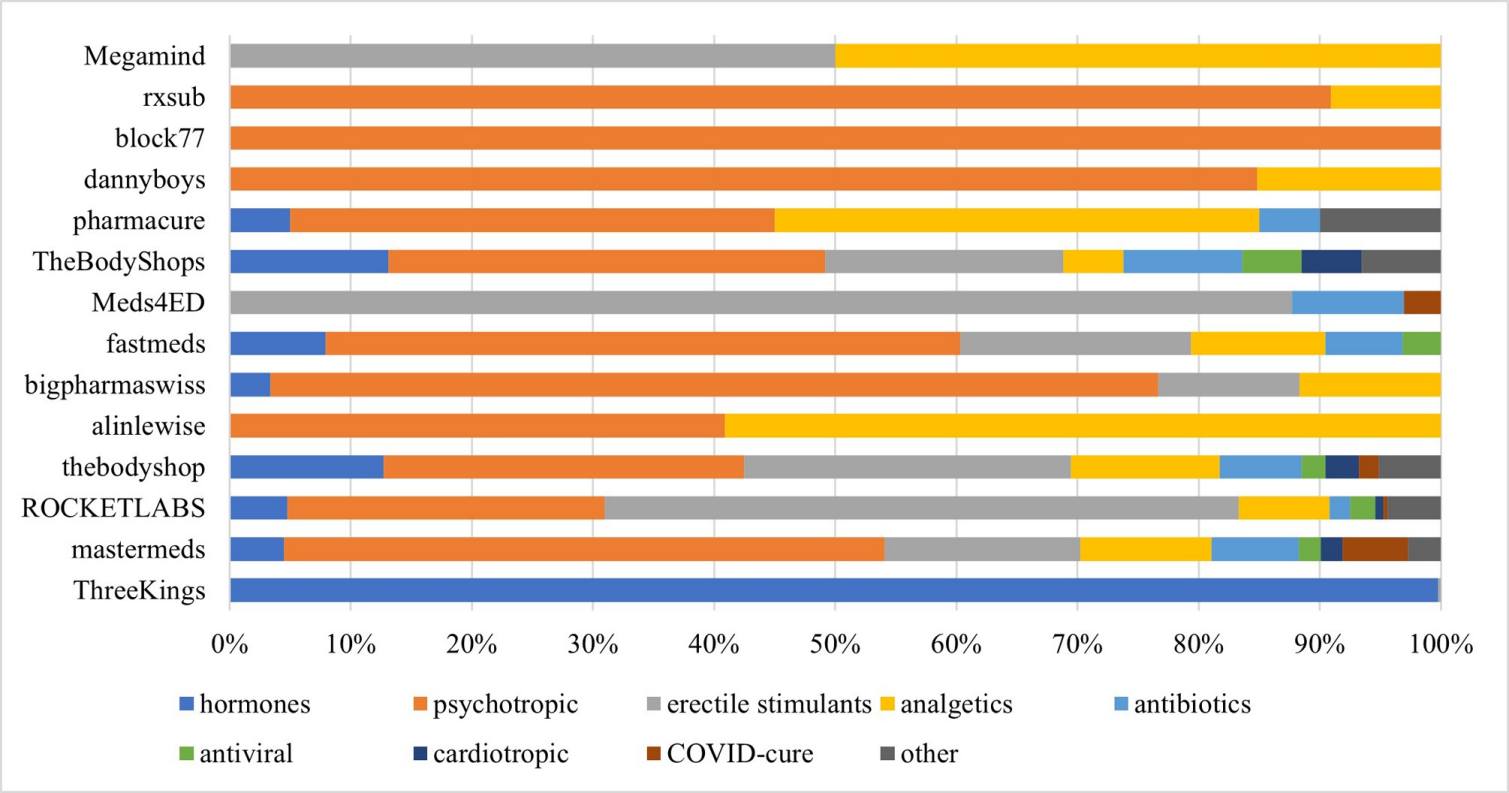


Among advertised medications, the most prevalent were hormones (steroid and thyroid)– 911 items (49%), psychotropic medications 371 (20%) and drugs against erectile dysfunction– 331 (19%). Fig 2 depicts the structure of medication subgroups for 14 vendors.

The correlation analysis revealed associations between COVID-19 and non-COVID-19 products. The COVID-19 vaccines were more likely to be sold by vendor specializing in weapons (r = 0.83, p<0.001), money (r = 0.52, p = 0.014) and technologies (r = 0.45, p = 0.037); while COVID-cures were offered by those with commercial interests in general medications (r = 0.63, p = 0.002). COVID-medications were significantly less likely to be offered by the vendors dealing with documentations (r = -0.44, p = 0.04) (Table 5).

**Table 5:** Correlational matrix of the COVID-19 and non-COVID-19 product types.

COVID-19-related products	Non-COVID-19-related products (r, p-value)					Number of product types
	Medications	Money	Weapons	Technologies	Documents	
COVID-cards	-0.66, 0.001	0.11, 0.64	-0.32, 0.15	0.002, 0.99	0.09, 0.69	-0.08, 0.72
COVID-vaccines	-0.13, 0.58	0.52, 0.014	0.83, <0.001	0.45, 0.04	0.37, 0.09	0.47, 0.03
COVID-cure	0.63, 0.002	-0.28, 0.20	-0.02, 0.92	-0.19, 0.40	-0.44, 0.04	-0.19, 0.39
COVID-tests	0.40, 0.68	-0.29, 0.19	-0.16, 0.48	-0.13, 0.58	0.06, 0.79	-0.5, 0.81

Among the non-COVID-related items, financial services were more likely to be proposed in association with weapons (r = 0.60, p = 0.003) and hacking (r = 0.63, p = 0.002).

There were several correlates between COVID “cure” and particular medications (Table 6). For instance, COVID-cures were more often provided by the vendors specialising in antibiotics, erectile stimulants, cardiotropic and antiviral drugs.

**Table 6:** Correlations between COVID-cure and other medication groups.

Medication groups	r	p-value
Hormones	0.33	0.254
Psychotropic	0.44	0.116
**Erectile stimulants**	**0.77**	**0.001**
Analgesics	0.37	0.188
**Antibiotics**	**0.80**	**0.001**
**Antiviral**	**0.55**	**0.040**
**Cardiotropic**	**0.63**	**0.015**
Other	**0.59**	**0.027**

## Discussion

This study is one of the first to identify the availability of COVID-19 vaccines, fake test certificates and hypothetical/illegal cures across DWMs with a methodology which enabled a retrospective analysis of the darkweb content. For the products identified online, a correlation with the time frame of the pandemic milestones was identified. Moreover, the correlation between vendors’ portfolios of COVID-19 products and variety of other illicit goods, such as illegal weaponry, medication/drugs of abuse has been highlighted.

Overall, 42 listings of unlicenced COVID-19 products emerged from our searches for the period between March 2020 and October 2021. Products on offer were found to be time specific according to the unfolding of the pandemic (Fig 1), in line with what reported by Bracci et al. [16, 18]. For instance, all the retrieved “certificates” [13] were advertised for sale after August 2021 when individuals started travelling again after the lockdown [12, 19, 20], with only one being advertised in June 2021 (Table 2). Sold among a plethora of other fake documents, they varied from simple blank cards to supposedly “official” vaccination certificates authorised by healthcare professionals [21]. They were made available for shipment in various countries including Europe, USA and Canada.

COVID-19 “cures” were first advertised on the dark net in late 2020 in the form of Hydroxychloroquine 100 mg (Table 2) as a result of some media and scientific claims supporting its possible activity against COVID-19 symptoms [22, 23]. Once these claims were found inaccurate [24, 25], Hydroxychloroquine was quickly replaced by another two products IVERMECTIN and Amantadine (2021), with the latter gaining popularity as an aid to tackle the COVID-19 outbreak in India and Brazil [26–29]. Both were advertised in 2020 and 2021 despite the declared ban applied by some DWMs on the advertisement and sale of legitimate/illicit vaccines. Although DWMs have sometimes a “code of conduct” and specific regulations against the sale of very harmful or immoral items (e.g., potent drugs of use/abuse, some types of pornography) [30–32], the latter had no effect in this case. Remarkably, there are currently no effective strategies for preventing and deterring individuals from buying harmful products from the DWMs. These include those seeking drugs for recreational purposes, patients who are lacking both financial resources and health insurance wanting to purchase prescription drugs, sometimes (but not exclusively) to control pain as well as individuals who then resell the products on the street market [33] In the case of our study, the offer of fake certifications and COVID-19 vaccines seemed to be aimed predominantly at those who preferred not to be vaccinated, but still needed a certification of vaccination for mobility, work, social and other reasons. On the other hand, “cures” for the COVID-19 seemed to target a more vulnerable cohort including individuals from countries where it was more difficult to manage timely health responses to the pandemic. Indeed, it is worth noting that effective vaccination campaigns and health care support have been mainly available in few and often wealthy countries, while the rest of the world (with very few exceptions like Cuba) was left behind due to insufficient vaccines stocks and inadequate infrastructures. Their contact with unscrupulous vendors selling a variety of other illicit goods, such illicit drugs, weapons, human organs among others, make their behaviour even more hazardous [34, 35].

Further, the identification of a specific timeline for each product’s appearance on the DWMs confirmed the very flexible and dynamic nature of such markets and their ability to follow external trends and adapt to the everchanging global supply and demand. We noticed that new criminal businesses became ‘active’ on demand when a new profit opportunity related to the pandemic emerged regardless of any pre-existing expertise or commercial experience (e.g., handling, distribution, sale). The fact that some of the 24 identified vendors displayed very small portfolios (with 1 or two COVID-19 related items) confirms the versatile and opportunistic nature of some actors in DWMs. As also emerged from Bracci et al., vendors often do not provide complete information on their listings but rather invite direct communication to facilitate sales. However, the larger part of the identified vendors were well established sellers displaying large and diversified portfolios (Table 4). We also found a strong correlation between the COVID-19 related products and the sale of medications, licit or counterfeit, while vaccines and certificates were offered by those who traded fake currencies, weapons, counterfeit identity documentation and cyber frauds. These might have well been operated by large, organised crime groups rather than individuals.

In terms of methodologies, the innovative scraping techniques we used unable us to access data on both live/defunct marketplaces and active/unactive online vending sites, which would have not been possible via Tor or other standard DWM browsers. This reinforces the importance of using an intelligence-led approach to better target DWMs and vendors generating the greatest levels of risk or harm, which in turn may generate the greatest crime and harm reduction returns. Such efforts might be well combined with prevention and demand reduction activities aimed at educating and informing those purchasing products from these illicit marketplaces about the inherent risks and uncertainties of doing so. Buyers of the identified COVID-19 products, possibly led by misleading claims, have engaged in unethical, harmful, and irresponsible behaviours for themselves and society at large. In fact, it has been demonstrated that the severity of COVID-19 is greater in non-vaccinated individuals, with an associated increase in the transmissibility of the virus [36]. To prevent such unwanted harmful consequences, further monitoring, and regulatory responses of DWMs should be considered to protect the health and safety of citizen especially at times of global crisis.

### Limitations

This study has a few limitations; however, all efforts were made to minimise these throughout.

The data collected is restricted to that which has been seen and processed by the utilised Cybersolace platform, however this data-collection method has previously been successfully utilised by this research group [34].

The limited sample size collected means generalisations about availability based on this data are restricted. However, as this research was largely exploratory and intended to identify the initial nature and extent of COVID-19 related products on DWMs with a view to informing policy response, we feel the insights generated are of value.

Furthermore, this study did not purchase any products advertised for sale on the observed DWM listings and so cannot testify the genuineness of the products listed available, nor their actual availability.

Furter analysis online on COVID-19 related products will involve the investigation of the correlation between the latter and the COVID-19 mortality or vaccine injection trends.

## Conclusion

The identified availability and easy accessibility of a wide range of COVID-19 related products of illicit nature across 118 DWMs markets raise important health and safety concerns due both hazardous voluntary consumption and potential harm to others.

Considering the present COVID-19 scenario worldwide, it is expected that claimed cures, vaccines and certificates will keep being advertised on DWMs for the foreseeable future. However, the forms that these product-types take may be subject to change as requirements adjust around the world. In particular, new oral antiviral products (e.g., molnupiravir, nirmatrelvir and ritonavir) are currently being approved worldwide for treatment of mild to moderate COVID-19 infections [37, 38] and the future accessibility of these products will likely impact on their evolving availability on DWMs.

The results from this study point to the importance of using an intelligence-led approach to better target resources at those DWMs and vendors generating the greatest levels of risk or harm (which in turn may generate the greatest crime and harm reduction returns on this investment). This more targeted approach should continue to form part of a balanced and integrated DWM strategy which includes complimentary prevention and demand reduction activities aimed at educating and informing those purchasing products from these illicit marketplaces about the inherent risks and uncertainties of doing so.

It is important to continue monitoring these DWMs in order to assess the continuing sale of COVID-19 related products and to continually inform policy makers on the potential health risks connected to or posed by the sale of these products. Information campaigns targeted to DWMs’ potential clients and more vulnerable cohorts of people should be designed and disseminated, to discourage the purchase of such items. As reported by INTERPOL and the United States’ Homeland Security Investigations (HSI), “the risks to the public are clear: these can include buying a product which not only does not protect against COVID-19 but poses a serious health hazard if ingested or injected. Such products are not tested, regulated or safety-checked” [39].
