# Supplementary material for: Illicit COVID-19 products online: A mixed-method approach for identifying and preventing online health risks
Source: PLoS One. 2023 Jun 16;18(6):e0287231. doi: 10.1371/journal.pone.0287231 (PMC10275425; doi:10.1371/journal.pone.0287231)
Supplement: Peer review history

# Peer review: Decision Letter 0

**DOI:** 10.1371/journal.pone.0287231.r001

**Authors:** Mohammad-Reza Malekpour

13 Dec 2022

PONE-D-22-26584Illicit COVID-19 products online: Identify and preventing online health risksPLOS ONE

Dear Dr. Catalani,

Thank you for submitting your manuscript to PLOS ONE. After careful consideration, we feel that it has merit but does not fully meet PLOS ONE’s publication criteria as it currently stands. Therefore, we invite you to submit a revised version of the manuscript that addresses the points raised during the review process.

==============================The subject of the manuscript is understudied, influential, and interesting to the scientific community; however, there are some points that need to be addressed before publication. One of the main issues with the manuscript is the way it is presented and its structure. Please implement a solid storyline and combine separated islands of information into paragraphs. Another suggestion is performing time-series analysis and comparing the trends and peaks with other aspects of COVID-19, such as infodemics vs. scientific publications (https://doi.org/10.1371/journal.pone.0258064).

==============================

Please submit your revised manuscript by Jan 27 2023 11:59PM. If you will need more time than this to complete your revisions, please reply to this message or contact the journal office at plosone@plos.org. When you're ready to submit your revision, log on to https://www.editorialmanager.com/pone/ and select the 'Submissions Needing Revision' folder to locate your manuscript file.

Please include the following items when submitting your revised manuscript:

- A rebuttal letter that responds to each point raised by the academic editor and reviewer(s). You should upload this letter as a separate file labeled 'Response to Reviewers'.
- A marked-up copy of your manuscript that highlights changes made to the original version. You should upload this as a separate file labeled 'Revised Manuscript with Track Changes'.
- An unmarked version of your revised paper without tracked changes. You should upload this as a separate file labeled 'Manuscript'.

If you would like to make changes to your financial disclosure, please include your updated statement in your cover letter. Guidelines for resubmitting your figure files are available below the reviewer comments at the end of this letter.

If applicable, we recommend that you deposit your laboratory protocols in protocols.io to enhance the reproducibility of your results. Protocols.io assigns your protocol its own identifier (DOI) so that it can be cited independently in the future. For instructions see: https://journals.plos.org/plosone/s/submission-guidelines#loc-laboratory-protocols. Additionally, PLOS ONE offers an option for publishing peer-reviewed Lab Protocol articles, which describe protocols hosted on protocols.io. Read more information on sharing protocols at https://plos.org/protocols?utm_medium=editorial-email&utm_source=authorletters&utm_campaign=protocols.

We look forward to receiving your revised manuscript.

Kind regards,

Mohammad-Reza Malekpour

Academic Editor

PLOS ONE

Journal Requirements:

When submitting your revision, we need you to address these additional requirements.

1. Please ensure that your manuscript meets PLOS ONE's style requirements, including those for file naming. The PLOS ONE style templates can be found at 

https://journals.plos.org/plosone/s/file?id=wjVg/PLOSOne_formatting_sample_main_body.pdf and 

https://journals.plos.org/plosone/s/file?id=ba62/PLOSOne_formatting_sample_title_authors_affiliations.pdf.

2. Thank you for stating the following in the Acknowledgments Section of your manuscript: 

"The project was funded by the National Research Centre on Privacy, Harm Reduction and Adversarial Influence Online (REPHRAIN)."

We note that you have provided funding information that is not currently declared in your Funding Statement. However, funding information should not appear in the Acknowledgments section or other areas of your manuscript. We will only publish funding information present in the Funding Statement section of the online submission form. 

Please remove any funding-related text from the manuscript and let us know how you would like to update your Funding Statement. Currently, your Funding Statement reads as follows: 

"The project was funded by the National Research Centre on Privacy, Harm Reduction and Adversarial Influence Online (REPHRAIN) and Ornella Corazza was the principal investigator and received the funds"

Please include your amended statements within your cover letter; we will change the online submission form on your behalf.

[Note: HTML markup is below. Please do not edit.]

Reviewers' comments:

Reviewer's Responses to Questions

**Comments to the Author**

1. Is the manuscript technically sound, and do the data support the conclusions?

The manuscript must describe a technically sound piece of scientific research with data that supports the conclusions. Experiments must have been conducted rigorously, with appropriate controls, replication, and sample sizes. The conclusions must be drawn appropriately based on the data presented. 

Reviewer #1: Yes

Reviewer #2: Yes

Reviewer #3: Partly

**********

2. Has the statistical analysis been performed appropriately and rigorously? 

Reviewer #1: No

Reviewer #2: I Don't Know

Reviewer #3: I Don't Know

**********

3. Have the authors made all data underlying the findings in their manuscript fully available?

The PLOS Data policy requires authors to make all data underlying the findings described in their manuscript fully available without restriction, with rare exception (please refer to the Data Availability Statement in the manuscript PDF file). The data should be provided as part of the manuscript or its supporting information, or deposited to a public repository. For example, in addition to summary statistics, the data points behind means, medians and variance measures should be available. If there are restrictions on publicly sharing data—e.g. participant privacy or use of data from a third party—those must be specified.

Reviewer #1: Yes

Reviewer #2: Yes

Reviewer #3: Yes

**********

4. Is the manuscript presented in an intelligible fashion and written in standard English?

PLOS ONE does not copyedit accepted manuscripts, so the language in submitted articles must be clear, correct, and unambiguous. Any typographical or grammatical errors should be corrected at revision, so please note any specific errors here.

Reviewer #1: Yes

Reviewer #2: Yes

Reviewer #3: Yes

**********

5. Review Comments to the Author

Please use the space provided to explain your answers to the questions above. You may also include additional comments for the author, including concerns about dual publication, research ethics, or publication ethics. (Please upload your review as an attachment if it exceeds 20,000 characters)

Reviewer #1: Overall assessment:

Corazza et al., have performed a cross-sectional web-based study to identify the availability of illicit COVID products across the DWMs and solicit appropriate regulatory measures and targeted interventions to control the illegal and unsafe market. I think the subject is of interest and understudied. Moreover, the authors are experienced in the field and it would be worthful to add their contribution to this topic to the body of literature. However, the study needs to be improved, especially in the results and discussion section.

Overall, I think the manuscript needs major revision.

Major Comments:

• I think the authors did not perform data analysis to its full aspects and extent. For instance, as the authors mentioned, the date that products were offered on the dark web was gathered as a variable. However, no time trend was explored to present the number of products offered on the dark web (especially vaccines and drugs) each month or day. Other public access databases and time trends on COVID-19, such as COVID-19 mortality or vaccine injection, could be compared with the time trend of dark web activity with regard to illicit COVID-19 products. This comparison may elucidate the causes of some increases and decreases in the dark web activity

• The discussion needs to clearly present what are the main results of the study and how they are interpreted. The result of this study should be compared with similar studies such as the two ones performed by Bracci et al. There may be studies that provide an estimate of the rate of people that ever bought their needed COVID-19 products from the dark web, and this should be mentioned.

• In the objective of the study, the authors stated that they want to solicit appropriate regulatory measures and targeted interventions. However, such measures and interventions are discussed only in a few sentences. The part should be expanded.

Minor Comments:

• The abstract’s method subsection does not provide some essential information. The sentence “the latter allows rapid and ….” Is not mandatory for this subsection. Instead, a brief description of the variables assessed and analyses performed is missed, which has made it difficult to follow the results subsection and correlation results.

• Please sort table 2 based on a variable to make it easier to follow. Sorting based on the product type may be suitable.

• Table 3 could be added to supplementary material.

Reviewer #2: Dear Authors,

Thank you for your efforts for unveiling dark web markets offering illicit Covid products for sale. It’s an important topic.

I have read your manuscript and ask you to revise your work, hopefully my remarks and suggestions will be beneficial:

General comments:

1. The project is great, congratulations!

2. As the consequence is hindered patient and consumer safety, I suggest you to consider who would be the customer of these markets. I assume the general population has no idea how to access DWMs, according it would be nice to mention how such products would reach patients and consumers.

3. Introduction and discussion: Misinformation and infodemic has proven close correlation with the number of Covid cases and the prevalence of links referring to illegal online medicine sellers on the surface web. See: https://pubmed.ncbi.nlm.nih.gov/34299920/

4. Methods: number of products offered was hypothesised to reflect the vendors ‘market activity’. What do you mean by market activity? Has any previous study aimed to asses volume of sales of dark web outlets?

5. Methods: Why 118 DWMs? Is there any estimate of the total number of vendors or markets in the DW? How representative is your study?

6. Methods and discussion: As no test purchase was made, we can only assume these vendors possess and sell the products. It is noted in the limitation for the study.

7. Results: Table 3 should be a supplement, please exclude from the manuscript text.

Specific comments

1. Abstract: Proprietary threat intelligence platform is not evident for the reader.

2. Methods; Searches were conducted during the fall of 2021. Please refer briefly how retrospective data was gathered covering the the period from March 2020 to October 2021? It’s mentioned in the discussion, however not in methodology.

3. Results: Two out of eight markets were inactive, wile all vendors were noted to be active. Please help the reader with resolving the seemingly contradictory finding. How can someone evaluate the content of a market which is not active?

4. Results: Regarding the correlation, it may be interesting to evaluate the relatedness of multiple factors. If possible, as limited number of vendors are available in the dataset.

5. Discussion: paragraph 3, Similar findings have been identified for websites selling medicines on the surface web. Please consider reflecting on these as the pattern is seemingly the same.

Thank you

The Reviewer

Reviewer #3: This paper aimed to identify the availability of illicit vaccines and cures across the dark web markets and solicit appropriate regulatory measures and targeted interventions to protect the safety and wellbeing of vulnerable individuals and the wider society. Considering the disruption of the supply and demand balance in the treatment market when faced with a pandemic in societies, legislation and strict supervision have a significant effect in controlling unrealistic treatments. Therefore, reporting what happened in previous pandemics is very important and useful for the future. I reviewed the paper, and I found it interesting, but I think the authors should revise the manuscript for a better presentation. My comments are as follows:

Major comments:

• The whole manuscript contains a lot of short paragraphs. Please merge the related paragraphs.

• The introduction part is not coherent. Please redesign the outline of the introduction in a way that is concise and useful, starting from the known and moving toward the unknown. Also, please avoid generalizations in the introduction and directly express the contents that are completely related to the subject of the study.

• As a scientific article, most of the presented results seem unnecessary, and on the other hand, there is no proper classification in the presentation of the results. Part of the results have been addressed to the vendors and their contribution, although they can be added as an appendix, but in a scientific article, the distribution, nature, and performance of vendors are more important than their names.

• Adding a table or figure about locations (countries) where those vendors can sell their products will enrich the paper. This should be categorized as some products, like digital certificates, do not limit to locations and can be delivered immediately, but some other products that should be delivered physically are limited to some locations.

• Please use a checklist for reporting in scientific papers. For example, using the STROBE checklist would be beneficial.

Minor comments:

• As this study is a descriptive study, it is recommended to add the target population of investigated markets to the aim of the study. Also, you should mention that all searches were carried out in the English language.

• Please use the Vancouver style for citations.

• All abbreviations used in each table should be defined in the caption of that table.

• Please discuss more possible and effective ways to prevent vendors from selling on the dark web in the discussion section.

• Limitations should be moved to the end of the discussion.

• Please revised the conclusion section. Some sentences, like the last sentence, are more suitable for the discussion section. The conclusion should be a conclusion of the findings of your study and what should be considered more, as now we found from this study.

**********

6. PLOS authors have the option to publish the peer review history of their article (what does this mean?). If published, this will include your full peer review and any attached files.

If you choose “no”, your identity will remain anonymous but your review may still be made public.

**Do you want your identity to be public for this peer review?** For information about this choice, including consent withdrawal, please see our Privacy Policy.

Reviewer #1: No

Reviewer #2: **Yes: **András Fittler

Reviewer #3: No

**********

[NOTE: If reviewer comments were submitted as an attachment file, they will be attached to this email and accessible via the submission site. Please log into your account, locate the manuscript record, and check for the action link "View Attachments". If this link does not appear, there are no attachment files.]

While revising your submission, please upload your figure files to the Preflight Analysis and Conversion Engine (PACE) digital diagnostic tool, https://pacev2.apexcovantage.com/. PACE helps ensure that figures meet PLOS requirements. To use PACE, you must first register as a user. Registration is free. Then, login and navigate to the UPLOAD tab, where you will find detailed instructions on how to use the tool. If you encounter any issues or have any questions when using PACE, please email PLOS at figures@plos.org. Please note that Supporting Information files do not need this step.

# Author comment: Author response to Decision Letter 0

**DOI:** 10.1371/journal.pone.0287231.r002

26 Apr 2023

Nelissa Duarte

Straive Editorial Assistant, 

  

Plosone

Dear Dr Nelissa Duarte

please find here enclosed the revised manuscript (‘Illicit COVID-19 products online: Identify and preventing online health risks). You will also identify below the list of queries of Reviewers 1, and 2 and how they have been dealt with. We very much hope that with these modifications the paper will now be considered worthy of publishing in your Journal. 

Reviewer 1 

we would like to thank you for the effort and time you dedicated to read and revise our manuscript we are grateful for the comments received. We rae relay happy that you find the paper share vital information. We have made every attempt to fully address these comments in the revised manuscript and we are confident that the changes made following your directions have improved the content and strengthened the quality of our manuscript

Reviewer: 

I think the authors did not perform data analysis to its full aspects and extent. For instance, as the authors mentioned, the date that products were offered on the dark web was gathered as a variable. However, no time trend was explored to present the number of products offered on the dark web (especially vaccines and drugs) each month or day. Other public access databases and time trends on COVID-19, such as COVID-19 mortality or vaccine injection, could be compared with the time trend of dark web activity with regard to illicit COVID-19 products. This comparison may elucidate the causes of some increases and decreases in the dark web activity

Authors: Thanks for your comment. We indeed agree that associating a time trend to the COVID-19 related listing identified could be very interesting. On this topic we included a small paragraph in the original manuscript (pag 8) as follows “The COVID-19 related products were advertised in specific time frames that followed the pandemic timeline (Figure 1) The majority of hits retrieved were advertised on the dark web in 2021. All the “certification” hits were advertised starting from June 2021 to October 2021, while dates associated with the “cure” results spanned a broader time space from April 2021 to July 2021. Only one “cure” was advertised earlier (October 2020). Self-tests for Covid-19 were advertised in 2020 only (July and November), while each of the two vaccines were advertised in July 2020 and April 2021”. Consistent with this, we also included in the original manuscript Figure 1 which indeed presented a timeline of pandemic related events in relation to the DWMs listings appearance/advertisement. Although not yet performed an analysis comparing COVID-19 mortality or vaccine injection trends could be very interesting but at the moment outside the scope of this article. We added a line in the limitations section on this regard “Furter analysis online on COVID-19 related products will involve the investigation of the correlation between the latter and the COVID-19 mortality or vaccine injection trends” 

Reviewer:

The discussion needs to clearly present what are the main results of the study and how they are interpreted. The result of this study should be compared with similar studies such as the two ones performed by Bracci et al. There may be studies that provide an estimate of the rate of people that ever bought their needed COVID-19 products from the dark web, and this should be mentioned.

Authors: Thanks for your comment. Consistent with this, the discussion has been modified to clearly presents what are the main results of the study and a comparison with Bracci et al papers. The changes are here reported “This study is one of the first to identify for the first time the availability of COVID-19 vaccines, fake test certificates and hypothetical/illegal cures across DWMs with a methodology which enabled a retrospective analysis of the darkweb content. For the products identified online, the correlation with the time frame of the pandemic milestones is reported. It also provides the analysis and a correlation between vendors’ portfolios of COVID-19 products and variety of other illicit goods, such as illegal weaponry, medication/drugs of abuse.……………………………………………….

Reviewer: 

In the objective of the study, the authors stated that they want to solicit appropriate regulatory measures and targeted interventions. However, such measures and interventions are discussed only in a few sentences. The part should be expanded 

Authors: Thanks for your very permanent comment. To address it we included the following in the Conclusion session “The results from this study point to the importance of using an intelligence-led approach to better target resources at those DWMs and vendors generating the greatest levels of risk or harm (which in turn may generate the greatest crime and harm reduction returns on this investment). This more targeted approach should continue to form part of a balanced and integrated DWM strategy which includes complimentary prevention and demand reduction activities aimed at educating and informing those purchasing products from these illicit marketplaces about the inherent risks and uncertainties of doing so.”

Reviewer: 

The abstract’s method subsection does not provide some essential information. The sentence “the latter allows rapid and ….” Is not mandatory for this subsection. Instead, a brief description of the variables assessed and analyses performed is missed, which has made it difficult to follow the results subsection and correlation results. 

Authors: Thanks for noticing that a more detailed description of the methodology was needed in the abstract. The latter has been reworded as follows “A retrospective search for COVID-19 related products was carried out across 118 DWMs at the start of the pandemic (March 2020-October 2021). Data on vendors as well as advertised goods such as asking price, marketplace, listed date were collected and further validated through additional searches on the open web to verify the information relating to specific marketplaces. Both quantitative and qualitative methods were used for data analysis.”

• Please sort table 2 based on a variable to make it easier to follow. Sorting based on the product type may be suitable. • Table 3 could be added to supplementary material 

Authors: Thanks for your comment. We proceeded to sort the table according to the product type as suggested and we moved table 3 to the supplementary material. 

Reviewer 2

we would like to thank you for the effort and time you dedicated to read and revise our manuscript, and we are grateful for the comments received. We have carefully attempted to address all the comments received and we do believe that following your directions and suggestion has strengthened and improved the content of the paper.

Reviewer: 

1. The project is great, congratulations!

2. As the consequence is hindered patient and consumer safety, I suggest you to consider who would be the customer of these markets. I assume the general population has no idea how to access DWMs, according it would be nice to mention how such products would reach patients and consumers. Ornella che qua puoi aggiungere qualcosa tu?

Authors: Thanks for your comment. We do agree it will be interesting to suggest who would the consumer of these markets. Hence we included a part to mention this in the discussion as follows “that may led to unwanted exposure and media/policy attention due to the dangerous/unethical nature of their trade (Hamill, 2015; IDPC, 2018; Townsend, 2018), it has had no effect in this case. Remarkably, there are currently no effective strategies for preventing and deterring vendors from selling harmful goods on DWMs”

…….

Reviewer: 

3. Introduction and discussion: Misinformation and infodemic has proven close correlation with the number of Covid cases and the prevalence of links referring to illegal online medicine sellers on the surface web. See: https://pubmed.ncbi.nlm.nih.gov/34299920/

Authors: Thanks for your comment and for suggesting this very interesting paper. Accordingly we added a sentence in the introduction as follows “In addition infodemic and misinformation correlated with the number of COVID-19 cases have been proven to increase the demand for alleged cures and the prevalence of related links on the surface web (Fittler et al 2021). The topic was however not considered in the discussion because despite extremely important, this topic was not investigates. Indeed the correlation on which this paper focuses is the timeline of the pandemic milestones ”……….”

Reviewer: 4. Methods: number of products offered was hypothesised to reflect the vendors ‘market activity’. What do you mean by market activity? Has any previous study aimed to asses volume of sales of dark web outlets?. 

Authors: Thanks for your comment. We agreed that the term market activity was unclear hence we removed it and proceeded to rewording the method section 

Reviewer: Why 118 DWMs? Is there any estimate of the total number of vendors or markets in the DW? How representative is your study?

Authors: Thanks so much for your comment. As reported in the method section the DW analysis was carried out via the use of the Cybersolace platform. These 118 DWMs were those currently (at the time of the study) monitored by the platform. A brief sentence has been added in the method section as follows “A total of 118 DWMs, i.e. those monitored by the platform, was used for the study. “ The authors, to the best of their knowledge are not aware of an estimation of the total vendors or markets in the DW. Moreover the dark web is a constantly evolving and shifting space, so any numbers or estimates about the number of markets present on it are likely to be inaccurate or outdated very quickly. We think that 118 DWMS, when compared to the similar literature ( Bracci et al, 2021 for example) could be considered fairly representative. Despite this, the fact that only the DWMs overlooked by Cybersolace were analysed is included in the limitation section. 

Reviewer: 6. Methods and discussion: As no test purchase was made, we can only assume these vendors possess and sell the products. It is noted in the limitation for the study. 

Authors: Thanks for your comments and for noticing this. We do agree with you that test purchase could be helpful in this kind of study and we are planning to include this type on analysis in future research project

Reviewer:7. Results: Table 3 should be a supplement, please exclude from the manuscript text 

Authors: Thanks for your comment, this table has been moved to supplementary material 

Reviewer 2 specific comments 

Reviewer: 1. Abstract: Proprietary threat intelligence platform is not evident for the reader. 

Authors: Thanks so much for your comment. We agreed that this could not be evident for the reader, however we could not add more info in the abstract due to the word limit. The concepts has been described in the methods section as follows “Digital trace data was collected from prominent darknet marketplaces, using a secured platform provided by the cybersecurity company CyberSolace, a private sector company specialising in information security advisory services. The platform is primarily used for threat intelligence and provides monitoring of the DWMs by performing rapid and reproducible searches for different market places, vendors, and items.”

Reviewer:2. Methods; Searches were conducted during the fall of 2021. Please refer briefly how retrospective data was gathered covering the the period from March 2020 to October 2021? It’s mentioned in the discussion, however not in methodology 

Authors: Thanks for noticing this. The relating part has been changed accordingly as follows “The ‘search spiders’ developed using Python scripts allow a rapid scanning of the darknet as well as a retrospective analysis of the marketplaces which no longer exist due to law enforcement operation or exit scams. This is an advantage compared to Tor or other traditional browsers which are unable to detect activities of markets that are no longer available.

Searches were conducted in October 2021 and covered the period from March 2020 to October 2021.”

Reviewer: 3. Results: Two out of eight markets were inactive, wile all vendors were noted to be active. Please help the reader with resolving the seemingly contradictory finding. How can someone evaluate the content of a market which is not active? 

Authors: Thanks for noticing this seemingly contradictory finding. We need to clarify that vendors usually sell/advertise across multiple DWMs, hence there is not a direct correlation between markets activity/inactivity and sellers activity/inactivity. As per our previous suggestion we added a sentence in the method section suggesting the capability of the platform to analyse market which were no longer active at the time of the study. However, in the results section we do not comment on the issue, hence a sentence has been included as follows “The analysis of the content of inactive markets was possible, as mentioned in the method section thanks to the nature of the scraping platform. “

Reviewer: Results: Regarding the correlation, it may be interesting to evaluate the relatedness of multiple factors. If possible, as limited number of vendors are available in the dataset 

Authors: Thanks so much for your comment. During the analysis, we retrieved all available data from the Platform that included the name of vendors, prices, delivery routes and ways. Indeed, as reviewer underlined, that could be of great importance to investigate the correlations not only product types but also prices and vendor characteristics. Unfortunately, the information on each vendor was not exhaustive and omit prices, delivery routes in many cases. Considering this, the authors were able to focus on the product types because the name of the products were inclusion criteria for the vendor analysis. 

Reviewer: Discussion: paragraph 3, Similar findings have been identified for websites selling medicines on the surface web. Please consider reflecting on these as the pattern is seemingly the same. 

Authors: Thanks for you comment. We agreed that similar findings have been reported on website selling on the surface web, but the comparison with the latter despite extremely interesting is at the moment outside the scope of this paper 

Reviewer 3

we would like to thank you for the effort and time you dedicated to read and revise our manuscript, and we are grateful for the comments received. We have carefully attempted to address all the comments received and we do believe that following your directions and suggestion has strengthened and improved the content of the paper.

Reviewer: Major comments:

• Reviewer The whole manuscript contains a lot of short paragraphs. Please merge the related paragraphs 

Authors: Thanks so much for your comment the paragraph have been merged 

• Reviewer The introduction part is not coherent. Please redesign the outline of the introduction in a way that is concise and useful, starting from the known and moving toward the unknown. Also, please avoid generalizations in the introduction and directly express the contents that are completely related to the subject of the study. 

Authors: Thanks for your comment. The introduction has been restructured and generalisation removed. 

• Reviewer As a scientific article, most of the presented results seem unnecessary, and on the other hand, there is no proper classification in the presentation of the results. Part of the results have been addressed to the vendors and their contribution, although they can be added as an appendix, but in a scientific article, the distribution, nature, and performance of vendors are more important than their names.

Authors Thanks a lot for your comment. As described in the limitations in this article we presented only those data which has been seen and processed by the utilised Cybersolace platform. These unfortunately are limited and do not include distribution, nature, and performance of vendors. However, this data-collection method has previously been successfully utilised by this research group and was deemed suitable for this project. 

• Adding a table or figure about locations (countries) where those vendors can sell their products will enrich the paper. This should be categorized as some products, like digital certificates, do not limit to locations and can be delivered immediately, but some other products that should be delivered physically are limited to some locations. 

Authors: Thanks so much for your comments. We added a column in Table 3 to indicate the location in/for which the product listing was intended or shipped. We agree on the fact that delivery might be more difficult or would be location/region limited. But this could also apply to certificate due to their country specific form/type. 

• Please use a checklist for reporting in scientific papers. For example, using the STROBE checklist would be beneficial. 

Authors: Thanks so much for your useful comment, the manuscript has been revised according to the STROBE checklist. Strobe checklist have been used as guidelines.

Minor comments:

•As this study is a descriptive study, it is recommended to add the target population of investigated markets to the aim of the study. Ornella can you please help here

Authors: Thanks so much for your comment, following which we revised the discussion session as follows “Remarkably, there are currently no effective strategies for preventing and deterring individuals from buying harmful products from the DWMs. These include those seeking drugs for recreational purposes, patients who are lacking both financial resources and health insurance wanting to purchase prescription drugs, sometimes (but not exclusively) to control pain as well as individuals who then resell the products on the street market (Aldridge et al 2016). In the case of our study, the offer of fake certifications and Covid-19 vaccines seemed to be aimed predominantly at those who preferred not to be vaccinated, but still needed a certification of vaccination for mobility, work, social and other reasons. On the other hand, “cures” for the Covid-19 seemed to target a more vulnerable cohort including individuals from countries where it was more difficult to manage timely health responses to the pandemic. Indeed, it is worth noting that effective vaccination campaigns and health care support have been mainly available in few and often wealthy countries, while the rest of the world (with very few exceptions like Cuba) was left behind due to insufficient vaccines stocks and inadequate infrastructures. Their contact with unscrupulous vendors selling a variety of other illicit goods, such illicit drugs, weapons, human organs among others, make their behaviour even more hazardous (Negri et al 2021; RAND 2016).”

Also, you should mention that all searches were carried out in the English language. 

Authors. Thanks for your comment this is mentioned in the methods section as follows “All searches were carried out in the English language “

 Please use the Vancouver style for citations. All abbreviations used in each table should be defined in the caption of that table.• 

Authors. Thanks for your comments. The reference style has been switched to Vancouver and all the abbreviation listed in each table defined in the caption. 

Please discuss more possible and effective ways to prevent vendors from selling on the dark web in the discussion section. 

Authors: Thanks for your very interesting comments. A brief discussion on possible and effective ways to prevent vendors activity on the darkweb was introduced in the discussion as follows “We know remarkably little about effective strategies for preventing and deterring vendors from selling on DWMs. Emerging evidence in related fields (Leclerc et al., 2021), however, points to the importance of developing crime scripts to better understand how different actors operate within and between DWMs, and the need to enhance and develop law enforcement capacity to prevent, detect and investigate illicit activities on these platforms. “

• Limitations should be moved to the end of the discussion. 

Authors. Thanks for your comment. The Limitations have be moved to the end of the discussion.

• Please revised the conclusion section. Some sentences, like the last sentence, are more suitable for the discussion section. The conclusion should be a conclusion of the findings of your study and what should be considered more, as now we found from this study. 

Authors: Thanks for noticing this. The conclusion has been reworded accordingly 

Finally, a full and thorough editing of the whole paper has been carried out, given the changes which have been here kindly suggested by the Reviewers.

Thanks again for taking the time to review our manuscript.

Best Regards

Dr V Catalani, on behalf of all the Authors

Attachment Submitted filename: Reviewers letter_April.docx Click here for additional data file.

# Peer review: Decision Letter 1

**DOI:** 10.1371/journal.pone.0287231.r003

**Authors:** Mohammad-Reza Malekpour

2 Jun 2023

Illicit COVID-19 products online: a mixed-method approach for identifying and preventing online health risks

PONE-D-22-26584R1

Dear Dr. Catalani,

We’re pleased to inform you that your manuscript has been judged scientifically suitable for publication and will be formally accepted for publication once it meets all outstanding technical requirements. Although minor comments of reviewer 2 are not mandatory to be addressed, we have included them in this decision letter.

Within one week, you’ll receive an e-mail detailing the required amendments. When these have been addressed, you’ll receive a formal acceptance letter and your manuscript will be scheduled for publication.

An invoice for payment will follow shortly after the formal acceptance. To ensure an efficient process, please log into Editorial Manager at http://www.editorialmanager.com/pone/, click the 'Update My Information' link at the top of the page, and double check that your user information is up-to-date. If you have any billing related questions, please contact our Author Billing department directly at authorbilling@plos.org.

If your institution or institutions have a press office, please notify them about your upcoming paper to help maximize its impact. If they’ll be preparing press materials, please inform our press team as soon as possible -- no later than 48 hours after receiving the formal acceptance. Your manuscript will remain under strict press embargo until 2 pm Eastern Time on the date of publication. For more information, please contact onepress@plos.org.

Kind regards,

Mohammad-Reza Malekpour

Academic Editor

PLOS ONE

Reviewers' comments:

Reviewer's Responses to Questions

**Comments to the Author**

1. If the authors have adequately addressed your comments raised in a previous round of review and you feel that this manuscript is now acceptable for publication, you may indicate that here to bypass the “Comments to the Author” section, enter your conflict of interest statement in the “Confidential to Editor” section, and submit your "Accept" recommendation.

Reviewer #1: All comments have been addressed

Reviewer #2: All comments have been addressed

Reviewer #3: All comments have been addressed

**********

2. Is the manuscript technically sound, and do the data support the conclusions?

The manuscript must describe a technically sound piece of scientific research with data that supports the conclusions. Experiments must have been conducted rigorously, with appropriate controls, replication, and sample sizes. The conclusions must be drawn appropriately based on the data presented. 

Reviewer #1: Yes

Reviewer #2: Yes

Reviewer #3: Yes

**********

3. Has the statistical analysis been performed appropriately and rigorously? 

Reviewer #1: Yes

Reviewer #2: I Don't Know

Reviewer #3: I Don't Know

**********

4. Have the authors made all data underlying the findings in their manuscript fully available?

The PLOS Data policy requires authors to make all data underlying the findings described in their manuscript fully available without restriction, with rare exception (please refer to the Data Availability Statement in the manuscript PDF file). The data should be provided as part of the manuscript or its supporting information, or deposited to a public repository. For example, in addition to summary statistics, the data points behind means, medians and variance measures should be available. If there are restrictions on publicly sharing data—e.g. participant privacy or use of data from a third party—those must be specified.

Reviewer #1: Yes

Reviewer #2: Yes

Reviewer #3: Yes

**********

5. Is the manuscript presented in an intelligible fashion and written in standard English?

PLOS ONE does not copyedit accepted manuscripts, so the language in submitted articles must be clear, correct, and unambiguous. Any typographical or grammatical errors should be corrected at revision, so please note any specific errors here.

Reviewer #1: Yes

Reviewer #2: Yes

Reviewer #3: Yes

**********

6. Review Comments to the Author

Please use the space provided to explain your answers to the questions above. You may also include additional comments for the author, including concerns about dual publication, research ethics, or publication ethics. (Please upload your review as an attachment if it exceeds 20,000 characters)

Reviewer #1: Dear authors,

I would like to appreciate your efforts to revise the manuscript. I think my comments are addressed properly.

Regards,

Reviewer #2: Dear Authors,

Thank you for your thorough revision. I only have 2 comments now:

- Page 8: response to reviewer 1: please double check sentence on time frames, as June 2021 to October 2021 is broader than April 2021 to July 2021.

- Response to reviewer 2: Question no 2.: Accidentally a short comment was left in the text (“Ornella che qua puoi aggiungere qualcosatu?”). In line with the comment, I’m still missing information on who are the potential customers of DWMs. I have seen this topic was also mentioned by reviewer 3.

Kind regards

Reviewer #3: I would like to thank for the authors' diligent efforts in addressing the previous comments and making substantial improvements to the manuscript. After carefully reviewing the revised manuscript, I am pleased to acknowledge the extensive revisions you have made. It is evident that you have invested significant time and expertise in addressing the concerns raised by the reviewers. Your attention to detail and thoroughness in revising the manuscript are commendable.I think the manuscript now presents a well-structured and scientifically robust study that contributes significantly to the field. I believe that your work has the potential to make a valuable contribution to the scientific community.

**********

7. PLOS authors have the option to publish the peer review history of their article (what does this mean?). If published, this will include your full peer review and any attached files.

If you choose “no”, your identity will remain anonymous but your review may still be made public.

**Do you want your identity to be public for this peer review?** For information about this choice, including consent withdrawal, please see our Privacy Policy.

Reviewer #1: No

Reviewer #2: No

Reviewer #3: No

**********

# Editor report: Acceptance letter

**DOI:** 10.1371/journal.pone.0287231.r004

**Authors:** Mohammad-Reza Malekpour

7 Jun 2023

PONE-D-22-26584R1 

Illicit COVID-19 products online: a mixed-method approach for identifying and preventing online health risks 

Dear Dr. Catalani:

I'm pleased to inform you that your manuscript has been deemed suitable for publication in PLOS ONE. Congratulations! Your manuscript is now with our production department. 

If your institution or institutions have a press office, please let them know about your upcoming paper now to help maximize its impact. If they'll be preparing press materials, please inform our press team within the next 48 hours. Your manuscript will remain under strict press embargo until 2 pm Eastern Time on the date of publication. For more information please contact onepress@plos.org.

If we can help with anything else, please email us at plosone@plos.org. 

Thank you for submitting your work to PLOS ONE and supporting open access. 

Kind regards, 

PLOS ONE Editorial Office Staff

on behalf of

Dr. Mohammad-Reza Malekpour 

Academic Editor

PLOS ONE
